# Prevalence and antibiotic resistance of *Listeria monocytogenes* in camel meat

**DOI:** 10.1042/BSR20201062

**Published:** 2020-06-04

**Authors:** Hany M. Yehia, Manal F. Elkhadragy, Amani H. Aljahani, Khaloud M. Alarjani

**Affiliations:** 1 Food Science and Nutrition Department, College of Food and Agricultural Sciences, King Saud University, Riyadh 11451, Saudi Arabia; 2 Food Science and Nutrition Department, Faculty of Home Economics, Helwan University, Cairo, Egypt; 3 Biology Department, Faculty of Science, Princess Nourah bint Abdulrahman University, Riyadh 11671, Saudi Arabia; 4 Zoology Department, Faculty of Science, Helwan University, Cairo 11790, Egypt; 5 Department of Physical Sport Science, Nutrition and Food Science (PhD), Princess Nourah bint Abdulrahman University, Riyadh, P.O. Box 84428, Riyadh 11671, Saudi Arabia; 6 Department of Botany and Microbiology, College of Science, King Saud University, Riyadh 11451, Saudi Arabia

**Keywords:** antimicrobial resistance, Listeria monocytogenes, listeriolysin O (hly) gene, raw camel meat

## Abstract

In the present study, a total of 50 raw camel meat samples were analyzed for the presence of *Listeria monocytogenes.* The isolates were characterized via morphological and culture analyses; identification of isolates was confirmed by polymerase chain reaction (PCR) and sequencing of the listeriolysin O gene. The API *Listeria* system was used for further chemical identification and verification of the strains. *L. monocytogenes* was identified in eight raw camel meat samples, which was the highest incidence (16%) of contamination, followed by *L. seeligeri* 3(6%), *L. innocua* and *L. welshimeri* 2 (2% each), and *L. grayi* 1 (1%). According to Basic Local Alignment Search Tool (BLAST) analysis, isolated strains that were positive for the listeriolysin O gene were >99% similar to the published database sequences for *L. monocytogenes* strain LM850658 (sequence ID: CP009242.1). We studied the antibiotic resistance profile of the *L. monocytogenes* strains with common antibiotics used to treat human listeriosis and demonstrated that almost all strains tested were susceptible to the antibiotics.

## Introduction

*Listeria monocytogenes* is a Gram-positive rod-shaped bacterium that can cause human gastroenteritis [1] and is distributed widely in foods, particularly meat. *L. monocytogenes* can grow as a facultative anaerobe in psychrotrophic and mesophilic environments and can occasionally tolerate high temperatures. *L. monocytogenes* forms single, sometimes curved, coccobacilli, which may be connected in a chain and frequently in a ‘V’ shape; the bacterium is motile with peritrichous flagella that can be lost on entry into human cells. Filaments ranging in size from 6 to 20 μm may develop in aged cultures, which may also stain irregularly and have more filamentous shapes. Among the *Listeria* spp., which include *L. innocua, L. seeligeri, L. welshimeri, L. ivanovii*, and *L. grayi, L. monocytogenes* is considered as the only pathogenic species; however, some researchers have reported three cases of human infection caused by *L. ivanovii* [2,3] and one case of infection caused by *L. seeligeri* [4]. Nevertheless, among the six species, *L. monocytogenes* is the only species considered to have public health significance [5,6].

We focused on *L. monocytogenes* because of the multiple diseases that this species can cause, which include meningoencephalitis, meningitis, septicemia in immunocompromised individuals, newborns, and the elderly, and abortion and stillbirth in pregnant women [5,6]; systemic listeriosis is associated with a high rate of mortality, estimated to be between 20 and 40% [5]. Therefore, *L. monocytogenes* is considered to be one of the most important foodborne pathogens, and outbreaks and sporadic cases of listeriosis have been associated with meat and meat products [7]. Other bacterial pathogens known to contaminate these foods include *Escherichia coli* O157:H7 and *Campylobacter* spp. and these organisms have been linked to human illness [8,9].

In China, one of the first nationwide surveillances for the incidence of *L. monocytogenes* in meat products was supported by several provincial Chinese Centers for Disease Control and Prevention. Starting from 2010, the Chinese national monitoring network for microbial hazards in foods was established to survey all major foodborne pathogens in 31 provincial regions (i.e., provinces, autonomous regions, and municipalities) [10,11]. According to the Chinese literature published between 2011 and 2016, Li et al. [12] estimated that the overall *L. monocytogenes* prevalence of meat and poultry products (including raw and ready-to-eat products) was the highest (8.9%) among different food commodities. *Listeria* species are widespread in the environment, but only *L. monocytogenes* was considered to be an important human and animal pathogen [13].

Camel meat is considered to be a good source of nutrition and is similar in taste and texture to beef. Camel meat has an amino acid content that is ten-fold that of beef but has a lower intramuscular fat level [14]. In addition, antibiotics and hormones are not used at subtherapeutic or therapeutic doses in these animals unlike those used in other food animals. This suggests that microbiological flora in these animals may not be exposed to the same selective pressures as seen elsewhere in the meat industry [15]. It has been reported that *Listeria* spp. are susceptible to antibiotics active against Gram-positive bacteria; however, more recently, reports have indicated antibiotic resistance in *Listeria* spp. [16,17]. The increase in antibiotic resistance among *Listeria* spp. is in line with a general worldwide pattern of an increasing prevalence of antibiotic resistance, including multiple antibiotic resistances among many groups of bacteria; however, more information is required regarding the patterns of antibiotic resistance among *Listeria* spp.

Currently, there are limited studies on *L. monocytogenes* in camel meat in Saudi Arabia and its resistance to different antibiotics. Therefore, the aim of the present study is to investigate the incidence of *L. monocytogenes* in camel meat by identifying these organisms using polymerase chain reaction (PCR), using the listeriolysin O (*hly*) gene, and using the API LISTERIA system and to assess resistance to commonly used antibiotics.

## Materials and methods

### Camel meat samples

In the present study, a total of 50 samples of raw camel meat were purchased from local retail supermarkets located in Riyadh, Saudi Arabia. The samples were transported inside insulated cold boxes to the laboratory and analyzed on the same day.

### Detection and isolation of *L. monocytogenes*

*Listeria* species were isolated from the samples using cotton swabs and directly streaking on to HiCrome™ Listeria Agar Base, Modified (HIMEDIA M1417 (HiMedia Laboratories Mumbai, India)) supplemented with HiCrome Listeria Selective Supplement (FD181 (HiMedia Laboratories Mumbai, India)). The culture was incubated at 37°C for 24–48 h and compared with the standard strain of *L. monocytogenes* ATCC 19114. Those colonies morphologically resembling *Listeria* spp*.* were submitted for confirmatory examinations by Gram staining. Catalase test was carried out using 3% [m/m] hydrogen peroxide solution on glass slide. The appearance of bubbles indicates a positive catalase test. The bacteria were assessed by PCR using listeriolysin O gene primer forward (F) and reverse (R) genes as follows: F: 5′-ACTGAAGCAAAGG-′3, R: 5′-TTGGCGGCAC-3′.

### Genomic DNA extraction, PCR, and sequencing from isolated colonies

#### Extraction of genomic DNA

Genomic DNA was extracted from *L. monocytogenes* ATCC 19114 and listeria isolates using the G-spin™ Genomic DNA Extraction kit (iNtRON Biotechnology, Korea). Overnight cultures of *L. monocytogenes* ATCC 19114 and the other listeria isolates were grown in brain heart infusion (BHI) broth for 24 h at 37°C, after which 1–2 ml of cells (OD_600_ 0.8–1.0) were harvested by centrifuging at 13000 rpm for 1 min. The supernatant was removed, and the pelleted cells resuspended.

Lysozyme solution (3 μl in 50 μl of pre-buffer) was added to lyse the cells; the tube was inverted and mixed every 5 min during the incubation period. Then, 250 μl of G-buffer solution was added, and the mixture was inverted and mixed. The solution was removed, and 500 μl of washing buffer B was added to the column and centrifuged at 13000 rpm. The supernatant was removed, and the column was centrifuged for 1 min at 13000 rpm. The G-spin™ column was placed in a clean 1.5-ml microcentrifuge tube, and 50–200 μl of elution buffer was directly added on to the membrane. The loaded column was incubated at room temperature for 1 min, followed by centrifugation for 1 min. Two microliters of genomic DNA was loaded and electrophoresed on a 1% agarose gel and stained with Ethidium Bromide (5 μl); amplified DNA bands were visualized under ultraviolet light.

#### Detection of L. monocytogenes by listeriolysin O genes and PCR test

Total genomic DNA was isolated from *L. monocytogenes* ATCC 19114 and the listeria isolates using an AxyPrep™ bacterial genomic DNA miniprep kit (Axygen Scientific, Inc., U.S.A.) in accordance with the manufacturer’s instructions. Listeriolysin O gene primer.

For PCR, a 50-μl solution comprising 1× FIREPol® Master Mix Ready to Load (12.5 mM MgCl_2;_ Solis BioDyne, Tartu, Estonia), 2-μl listeriolysin O gene primer mix (50 pmol), 5-μl DNA template (50 μg/ml), and 33-μl of ultrapure water was used. DNA was amplified in a MULTEGENE thermal cycler (Labnet International, Inc. Edison, NJ) as follows: 95°C, 10 min; followed by 35 sequential cycles of 94°C for 1 min, 52°C for 1 min, 72°C for 1 min; and a final elongation step at 72°C for 10 min was performed after the completion of the cycles. The amplified PCR products, along with a 1-kb DNA ladder (GeneCraft), were separated on a 1.5% agarose gel (Sigma–Aldrich) containing Ethidium Bromide (0.5 mg/ml, ROTH) by electrophoresis (30 min at 100 V in 10× Tris-Borate-EDTA buffer; BIO Basic, Inc.), and visualized using a visual image analyzer software (Syngene).

#### Confirmation of L. monocytogenes identify by sequencing

Direct sequencing of the PCR products was performed to confirm the identity of the *hly* amplification product. The PCR products were purified and labeled using commercial kits according to the manufacturer’s instructions (AxyPrep™ PCR clean-up kit, Axygen®, NY, U.S.A.; BigDye Terminator v3.1 cycle sequencing kit, Applied Biosystems, CA, U.S.A.; BigDye X Terminator purification kit, Applied Biosystems, CA, U.S.A.) and as previously described by Al-Shabanah et al. [18]. The samples were sequenced using an automatic ABI 3500 genetic analyzer (Applied Biosystems, U.S.A.). Nucleotide sequences of 598 bp were identified by sequence alignment with the known sequences in the GenBank database using the Basic Local Alignment Search Tool (BLAST), provided by the National Cancer Institute, U.S.A.).

### Biochemical identification of *Listeria* spp

Listeria isolates were biochemically identified using the BioMérieux API *Listeria* system (BioMérieux, Marcy l’Etoile, France).

### Antibiotic profile for *Listeria* spp

The results of antimicrobial susceptibility tests of the *L. monocytogenes* strain ATCC 19114 and the *Listeria* isolates were compared. The tested bacterial isolates were obtained from overnight cultures following inoculation of single colonies into BHI media (Oxoid, U.K.). Cultures were then spread on Mueller Hinton agar (Oxoid, U.K.), and the individual plates were used for performing the agar disk diffusion experiment. A total of 16 different disks of antibiotics (Oxoid, U.K), belonging to 11 different classes were tested; these included β-lactam, polymyxin E, aminoglycosides, cyclic peptides, sulfonamide, macrobid, fluoroquinolone, oxazolidine, quinolone, chloramphenicol, and glycopeptide. The specific disks used were cephalothin (KF 30 μg, CT0010B), amoxicillin (AML 25 μg, CT0061B), ampicillin (AMP, 10 μg, CT0003B), cefoxitin (FOX, 30 μg, CT0119B), colistin (CT, 25 μg, CT0065B), polymyxin B (PB, 300 U, CT0044B), kanamycin (K, 30 μg, CT0025B), tetracycline (TE, 30 μg, CT0054B), sulfamethoxazole trimethoprim (SXT, 25 μg, CT0052B), nitrofurantoin (F, 300 μg, CT0036B), erythromycin (E, 15 μg, CT0020B), ciprofloxacin (Cip, 5 μg, CT0425B), linezolid (LZD, 30 μg, CT1650B), nalidixic acid (NA, 30 μg, CT0031B), chloramphenicol (C, 30 μg, CT0013B), and vancomycin (VA, 30 μg, CT0058B). The diameter of the zone of inhibition (mm) for each condition was determined using the criteria recommended for *L. monocytogenes* [19]. According to the diameter of the antibiotic inhibition zone, the tested isolates were classified as sensitive (S) or resistant (R) strains. Results were interpreted from the size of the inhibition zone.

## Results and discussion

### *Listeria* species confirmation and identification

#### *Listeria* colonies

**Figure 1:**
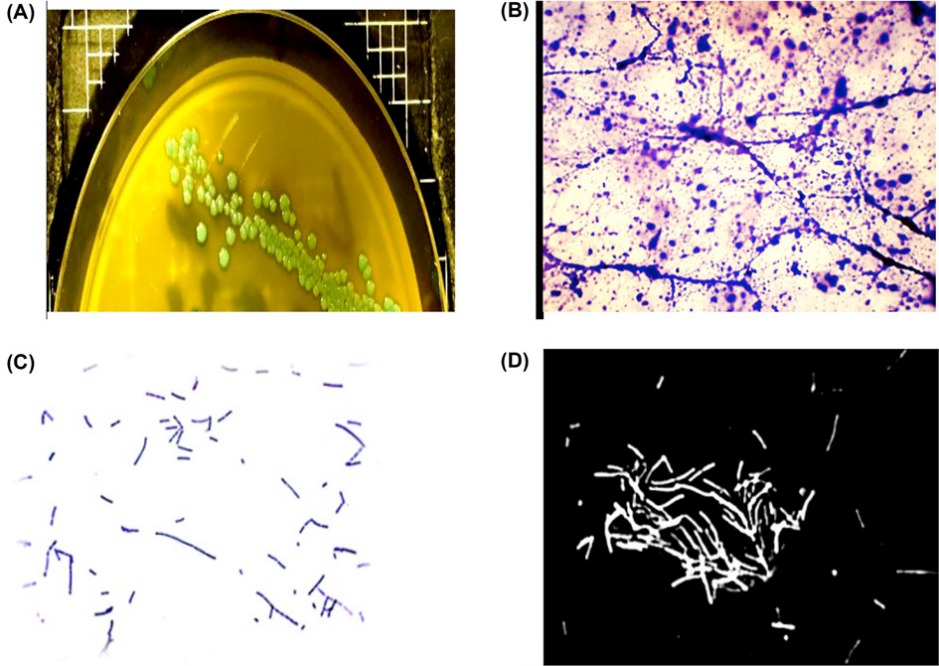
Morphological colony and microscopic examination of *L. monocytogenes* (**A**) Bluish-green colonies of *L. monocytogenes* on HiCrome™ Listeria Agar Base. (**B,C**) Staining of old and new cultures, respectively, with Gram stain and with (**D**) Nigrosine stain. Cultures were examined by light microscope (100×).

Identified *Listeria* spp. were cultured on HiCrome™ Listeria Agar Base, Modified (HIMEDIA M1417). Figure 1A, illustrates the appearance of *Listeria* spp. on *Listeria*-selective agar plates. This medium is based on the specific chromogenic detection of β-glucosidase activity as well as rhamnose fermentation. *Listeria* species hydrolyze the purified chromogenic substrate in the medium to produce blue-colored colonies. As β-glucosidase activity is specific to *Listeria* spp., other organisms cannot utilize the chromogenic substrate and therefore produce white colonies. Differentiation between *Listeria* spp. is based on the property of rhamnose fermentation. The colonies of *L. monocytogenes* appeared bluish-green with a yellow halo (rhamnose positive).

This medium also contains HiCrome™ Listeria Selective Supplement (FD181), which inhibits growth of most Gram-positive and Gram-negative bacteria as well as yeasts and molds [20]. Owing to the high salt tolerance of *Listeria* spp., lithium chloride was added to the medium. Medium containing esculin is used as listeria enrichment agar; esculin is a coumarin derivative extracted from the bark of flowering ash (*Fraxinus ornus*) and is a glycoside comprising glucose and a dihydroxycoumarin compound [20]. *Listeria* spp*.* hydrolyze esculin to the aglycone esculetin, which results in the formation of black iron phenolic compounds [20]. Thus, *L. monocytogenes* forms brown-green colored colonies with a black halo. Gram-negative bacteria are completely inhibited in all media plates, whereas Enterococci spp. grow poorly and exhibit a weak esculin reaction after 40 h of incubation at 37°C; however, some growth for a number of strains is indicated with brown colonies owing to esculin hydrolysis. Similarly, the growth of most Gram-positive bacterial species is suppressed.

***Gram staining***: Figure 1B shows that *Listeria* appeared as small short rods with rounded ends (0.5 μm in diameter and 1–2 μm or more in length). Older cells were observed as long filamentous bacteria in chains or as arranged in V and Y forms or palisades (Figure 1C).
***Nigrosine staining:*** The identification of *Listeria* species was confirmed through staining with Nigrosine dye, which causes the cells to appear long, thin, and with filamentous shapes (Figure 1D). Nigrosine is an acidic negative stain, this means that the stain readily gives up a hydrogen ion and becomes negatively charged, since the surface of most bacterial cells are negatively charged, and the cell surface repels the stain and appears clearly.
***Catalase test:*** Catalase production is an important test and thought to be a key virulence factor contributing to intracellular survival. The positive strain of *Listeria* can neutralizing the free radical killing effect of hydroxyl radicals formed within macrophages and other phagocytic cells during infection. In the catalase test, gas bubbles were produced in all of the tested samples after hydrogen peroxide was added to the suspension of each colony. The catalase enzyme serves to neutralize the bactericidal effects of hydrogen peroxide. Catalase expedites the breakdown of hydrogen peroxide (H_2_O_2_) into water and oxygen (2H_2_O_2_ + Catalase → 2H_2_O + O_2_). Also catalase was used to differentiate between aerobic and anaerobic bacteria.

### Identification of listeriolysin O gene in *L. monocytogenes* isolates

**Figure 2:**
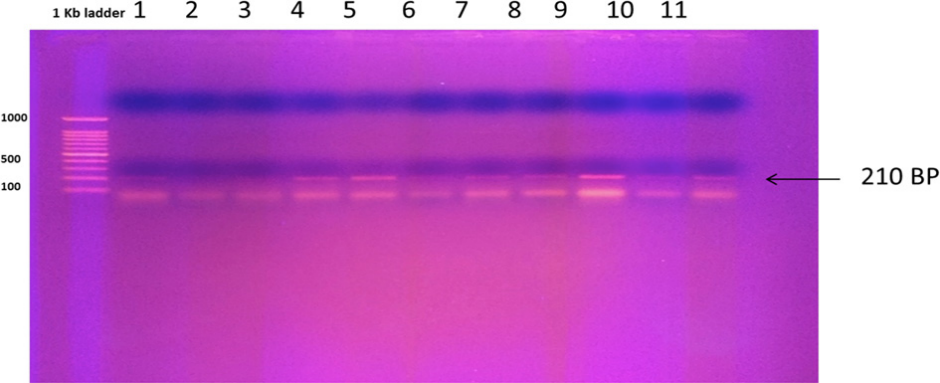
PCR product generated using genomic DNA and listeriolysin O gene primer Positive *hly* (listeriolysin O) amplification was present in *L. monocytogenes* ATCC 19114 (1) and in the eight isolates (4–11) at 210 bp; visualized by gel electrophoresis using 1% agarose with image analyzer (SYNGENE), DNA marker (1 kb ladder).

As shown in Figure 2, the band size observed in lanes 4–11 corresponded to a product of approximately 210 bp and was also present in the positive control, *L. monocytogenes* ATCC 19114 (lane 1). To verify that the product amplified was specific to the listeriolysin O gene, another PCR was run by using the *hly* gene primers and the PCR products from the three positive isolates (isolates 4, 5, and 9) and *L. monocytogenes* ATCC 19114 as templates under the same conditions. The results again demonstrated amplification of products at 210 bp and confirmed that the PCR products corresponded to the *hly* gene.

Direct DNA sequencing of the *Listeria* genomic DNA extracts using the listeriolysin O F primer as the forward sequencing primer obtained hypervariable regions unique to the *L. monocytogenes* strain. Submitting this 100-base pair (bp) sequence to GenBank for BLAST alignment analysis located the sequence to a region of the *L. monocytogenes* ATCC 19114 that was identified with the GenBank accession number JN703915.1; the three isolates (4, 5, and 9) were all identified with GenBank accession number CP009242.1) (Table 1).

### API *Listeria* system

The API *Listeria* test was incubated for 24 h and interpreted using the API LAB Plus bacterial computer identification program. Specifically, arylamidase (DIM test) results for all strains tested were unequivocal. Fermentation of carbohydrates resulting in acid production was interpreted according to change in pH color [21]. Data obtained from the 16 *Listeria* strains are shown in Table 2, and these patterns were used to rapidly identify *Listeria* spp. For arylamidase, α-mannosidase, glucose, and acid production from d-xylose, l-rhamnose, d-ribose, glucose-1-phosphate, and d-tagatose on an API *Listeria* strip was used for the accurate identification of *Listeria* spp. and subspecies identification. API *Listeria* tests conducted using API *Listeria* strips gave sufficient data for identification of *Listeria* species but did not give sufficient data regarding species identification, especially in the differentiation of *L. monocytogenes* from *L. innocua* [21]*.* This system does not require the use of additional tests, such as hemolysis on blood agar, and facilitates the genus identification and species determination of large numbers of microorganisms with minimal amounts of materials and labor and yields reliable results after a 24-h incubation period; furthermore, the API strip provides quick results and is inexpensive. Therefore, the API *Listeria* system may be a promising tool for routine use in many laboratories, particularly those concerned with food and environmental microbiology. Identification was carried out using numerical profiles for the positive samples within each group; a four-digit profile number was obtained using the API LISTERIA-IDENT databases (V4.1), and the analytical profile index as show in Table 2.

**Table 1:** DNA sequencing and identified according to GenBank for BLAST alignment analysis

	Partial sequences (∼100 bp)	Identification according to GenBank for BLAST alignment	Sequence ID
***L. monocytogenes* ATCC 19114**	CCAATCGAAAAGAAACACGCGGATGAAATCGATAAGTATATACAAGGATTGGATTATAATAAAAACAATGTATTAGTATACCACGGAGATGCAGTGACAAATGT	*Listeria monocytogenes* strain ATCC 19114 listeriolysin O (hly) gene, complete cds Sequence ID: JN703915.1 Length: 1590 Number of matches: 1	JN703915.1
**Isolate 4**	AAGTCCTAAGACGCCAATCGAAAAGAAACACGCGGATGAAATCGATAAGTATATACAAGGATTGGATTATAATAAAAACAATGTATTAGTATACCACGGAGATGCA	*L. monocytogenes strain* LM850658, complete genome Sequence ID: CP009242.1 Length: 2918502 Number of matches: 1	CP009242.1
**Isolate 5**	CGCCTGCAAGTCCTAAGACGCCAATCGAAAAGAAACACGCGGATGAAATCGATAAGTATATACAAGGATTGGATTATAATAAAAACAATGTATTAGTATA	*L. monocytogenes* strain LM850658, complete genome Sequence ID: CP009242.1 Length: 2918502 Number of	CP009242.1
**Isolate 9**	GCATCTCCGCCTGCAAGTCCTAAGACGCCAATCGAAAAGAAACACGCGGATGAAATCGATAAGTATATACAAGGATTGGATTATAATAAAAACAATGTAT	*L. monocytogenes* strain LM850658, complete genome Sequence ID: CP009242.1 Length: 2918502 Number of	CP009242.1

**Table 2:** Prevalence of *Listeria* species in raw camel meat and API *Listeria* identification

*Listeria* spp.	Number of isolates, *n*=50 (number of strains)	Four-digit numerical profile by API *Listeria* test	% ID
*L. monocytogenes*	8 (16)	6510	98.6
*L. innocua*	2 (4)	7410	99.2
*L. seeligeri*	3 (6)	3200	94.2
*L. grayi*	1 (2)	7020	99.9
*L. welshimeri*	2 (4)	1001	96.8
*Enterococcus sp.*	8 (16)	-	-
Not identified isolated	26 (52)	-	-

### The incidence of *Listeria spp.* in different samples

As shown in Table 2, *Listeria* spp. was recovered from 32% of the total tested samples (16 of 50 samples), *Enterococcus* sp., (16%), and other unidentified bacteria were 52%. *L. monocytogenes* constituted 50% of the *Listeria* spp., followed by *L. seeligeri* (18.75%), *L. innocua*, and *L. welshimeri* were both 12.5%, whereas *L. grayi* was 6.25%.

Studies on the prevalence and occurrence of *L. monocytogenes* in beef and meat products have been reported in several countries and have confirmed its contamination at all stages including processing to the final ready-to-eat products [22–25]. Consumer safety terms are used to explain that *Listeria* spp. are capable of growing in both raw and cooked meat at refrigeration temperature [26]. Many processes that transform raw meat into meat products can result in *L. monocytogenes* cross-contamination depending on the general hygienic and process parameters [27]. Yucel et al. [28] demonstrated that 79 (54.10%) of a total of 146 meat samples were contaminated with *Listeria* spp., with the highest incidence (86.4%) occurring in raw minced meat. *L. monocytogenes* was isolated from nine (6.16%) of the 79 samples examined in the present study; other species isolated included *L. innocua* in 68 samples (46.57%), *L. welshimeri* in one sample (0.68%), and *L. murrayi* in one sample (0.68%). Among the *Listeria* species, *L. innocua* (46.57%) was the most predominantly isolated species in the different varieties of meat samples. These results are in close agreement with the findings reported by Beak et al. [29] and Hudson et al. [30], who reported that the organism was recovered from 4.3 and 12.5% of meat samples, respectively. Abd El-Malek et. al., [31] reported that *L. innocua* in 83.3% of raw minced meat, 57.6% of raw chicken meat, 63.1% of beef, 9.6% of cooked red meat, and 10.7% of cooked chicken samples. This finding is in agreement with other studies where *L. innocua* was the most common species in raw and cooked meats, whereas other *Listeria* spp. were less frequent [32,33]. Yehia et al. [34] found that 50% of raw beef and chicken samples and 30% of raw fish samples contained listeria. In addition, *Listeria* spp. were found in 20% of the raw camel milk samples. Yehia et al. [34] explained that the samples with highest positivity were found to contain *L. seeligeri, L. welshimeri*, and *L. grayi* (*n*=3; 25%), followed by *L. monocytogenes* and *L. innocua* (*n*=2; 16.7%). In Brazil, 443 samples of listeria were assessed from different equipment, installations, and products from 11 meat-processing establishments [35]. These samples were analyzed using the USDA methodology for listeria detection, followed by species identification. The occurrence of listeria in the samples was 38.1%, of which 51.4% were obtained from equipment, 35.4% from installations, and 30.2% from products. The identified species were *L. monocytogenes* (12.6%), *L. innocua* (78.4%), *L. seeligeri* (1.2%), *L. welshimeri* (7.2%), and *L. grayi* (0.6%). The identified serotypes of *L. monocytogenes* were 1/2a and 4b. These results demonstrate the importance of equipment and installations as sources of listeria and *L. monocytogenes* contamination in the processing of beef and meat products.

### Antibiotic sensitivity of *Listeria spp.*

Table 3 summarizes the antimicrobial susceptibility profiles of *Listeria* spp. tested. The eight strains of *L. monocytogenes* LM850658 exhibited greater resistance than *L. monocytogenes* ATCC 19114 at 25 and 12.5%, respectively, from the 16 antibiotics tested. *L. monocytogenes* LM850658 was resistant to β-lactam class (KF 30 and FOX 30), and polymyxin E (CT 25) and quinolone (NA 30). *Listeria* have been reported to be naturally resistant to cephalosporins and fosfomycin [36,37] and these results agreed with that data. Generally, the overall incidence of antibiotic resistance in *L. monocytogenes* is relatively low in the present study. *L. innocua* ATCC 33090 is considered to be the most resistant *Listeria* spp. to wide classes of antibiotics tested than other *Listeria* spp. and was resistant to five classes of antibiotics, including β-lactam class (KF 30 and FOX 30), polymyxin E (CT 25 and PB 300), aminoglycoside (K 30), macrobid (F 300), and quinolone (NA 30); the percentage of resistance reached 43.75%. *L. innocua* 1 also had an overall resistance at 37.50%, followed b*y L. innocua* 2 at 18.75%. Our data are in agreement of [38] who reported that the level of resistance in *L. monocytogenes* was low (9.09%) compared with *L. innocua* (13.3%), *L. ivanovii* (25.0%), and *L. seeligeri* (50.0%).

**Table 3:** Antibiotic sensitivity of 24-h cultures of *Listeria* species and strains based on development of inhibitory zone diameters (mm) after application of disks containing specific antimicrobial agents

Strain No.	Antibiotic classes																Resistance %	Sensitivity %
	B-lactam				Polymyxin E		Aminoglycosides	Cyclic peptides	Sulfonamide	Macrobid		Fluoroquinolone	Oxazolidine	Quinolone	Chloramphenicol	Glycopeptide		
	KF 30	AMP 30	AML 25	FOX 30	CT 25	PB 300	K 30	TE 30	SXT 25	F 300	E 15	CIP 5	LZD 30	NA 30	C 30	V A 30		
***L. monocytogenes* ATCC 19114**	R	30	25	30	R	25	22	26	30	20	30	25	32	20	30	20	12.5	93.34
***L. monocytogenes* strain LM850658 (8 isolates)**	R	25	20	R	R	25	24	22	25	20	25	25	25	R	20	18	25	75
***L. innocua* ATCC 33090**	R	16	15	R	R	R	R	20	22	R	22	20	26	R	20	17	43.75	56.25
***L. innocua* 1**	R	30	24	R	R	R	R	20	25	20	20	24	24	R	22	20	37.50	62.50
***L. innocua* 2**	R	25	24	26	R	R	25	25	30	20	25	25	30	25	24	15	18.75	81.25
***L. seeligrei* 1**	R	30	30	25	R	R	20	27	30	24	24	25	22	25	17	25	18.75	81.25
***L. seeligrei* 2**	R	30	30	20	R	R	25	25	30	20	20	30	20	24	20	15	18.75	81.25
***L. seeligrei* 3**	R	30	30	2 0	R	R	30	25	30	20	25	26	25	20	20	20	18.75	81.25
***L. grey***	R	20	15	R	15	R	25	20	25	15	20	20	25	30	20	15	18.75	81.25
***L.* welshimeri 1**	20	17	17	15	15	R	25	20	20	17	20	20	20	15	17	15	6.25	93.75
***L.* welshimeri 2**	R	25	20	R	R	R	R	20	20	25	20	20	25	R	20	15	37.5	62.50

Zone of inhibition (mm); R = resistant; cephalothin (KF 30 μg, CT0010B), amoxicillin (AML 25 μg, CT0061B), ampicillin (AMP 10 μg, CT0003B), cefoxitin (FOX 30 μg, CT0119B), colistin (CT 25 μg, CT0065B), polymyxin B (PB 300 U, CT0044B), kanamycin (K 30 μg, CT0025B), tetracycline (TE 30 μg, CT0054B), sulfamethoxazole trimethoprim (SXT 25 μg, CT0052B), nitrofurantoin (F 300 μg, CT0036B), erythromycin (E 15 μg, CT0020B), ciprofloxacin (Cip 5 μg, CT0425B), linezolid (LZD 30 μg, CT1650B), nalidixic acid (NA 30 μg, CT0031B), chloramphenicol (C 30 μg, CT0013B), and vancomycin (VA 30 μg, CT0058B).

*L. seeligrei* 1, 2, and 3 were resistant to β-lactam class (KF 30) and polymyxin E (CT and PB 300), and the overall resistance was 18.75%. *L. grey* was also resistant to β-lactam antibiotics (KF 30 and FOX 30) and followed by polymyxin (PB 300), with a resistance ratio of 18.75%. *L.* welshimeri 2 was resistant to β-lactam class as (KF 30 and FOX 30), followed by polymyxin E (CT and PB 300), aminoglycosides (K 30), and quinolone (NA 30), and the ratio of resistance was 37.5%. *L.* welshimeri 1 was resistant only to polymyxin E (PB 300) with a resistant ratio of 6.25%. All *Listeria* isolates were sensitive to at least one antibiotic tested and sensitive to a specific class. Our data are in agreement with Wang et al. [38], who reported that the level of resistance in *L. monocytogenes* was lower (9.09%) than that in *L. innocua* (13.3%), *L. ivanovii* (25.0%), and *L. seeligeri* (50.0%). Our results also agree with those of Rota et al. [39] and Walsh et al., [16] indicating that a higher percentage of *L. innocua* strains were resistant to antibiotics than of *L. monocytogenes*. Stonsaovapak and Boonyaratanakornkit [40] stated that the level of resistance in *L. monocytogenes* was lower (5.6%) than that in *L. innocua* (16.0%), *L. ivanovii* (33.3%), and *L. seeligeri* (50.0%). No resistance was observed in *L. grayi* or *L. welshimeri.* Resistance to one antibiotic was more common than resistance to multiple drugs; only *L. innocua* showed resistance to multiple antibiotics, and it is noteworthy that *L. innocua* is known to carry resistance genes [41]. Generally, the ratio of resistance of strains in camel meat is not as prevalent as that of strains isolated from other meat products and may be because of the standard practice of feeding on natural pastures.

Yucel et al. (2005) [28], concluded that *Listeria* species isolated from raw (minced, chicken, beef) and cooked (red meat, chicken) meat samples were resistant to one or more antimicrobial agents (ampicillin, cephalothin, nalidixic acid). They suggest that the incorrect use of these antimicrobial agents for therapeutical purposes in veterinary science may lead to the development of antibiotic resistance.

Our results indicated that *Listeria* spp. susceptibility to antibiotics reached 100% with β-lactam (ampicillin and amoxicillin), followed by cyclic peptides (tetracycline), then sulfonamide as (sulfamethoxazole trimethoprim), macrobid (erythromycin), fluoroquinolone as ciprofloxacin, oxazolidine as linezolid, and chloramphenicol (chloramphenicol) and glycopeptide (vancomycin). These results are in agreement with published studies [39,42], which state that the *Listeria* genus was thought to be uniformly susceptible to antibiotics active against Gram-positive bacteria including ampicillin or penicillin (combined with aminoglycosides), trimethoprim (alone or combined with sulfamethoxazole), tetracyclines, erythromycin, and gentamicin. Hence, these antibiotics were used treatment of human listeriosis and veterinary medicine. Troxler et al. [43] reported high natural susceptibility among *Listeria* spp. to tetracycline, aminoglycosides, and chloramphenicol; however, a significant rate of resistant to cephalosporins was recorded. Similarly, Barbuti et al. [44] indicated that *L. monocytogenes* and *L. innocua* isolated from meat products had high sensitivity to chloramphenicol. Furthermore, Dhanashree et al. [45] also showed that *L. monocytogenes* was isolated with high sensitivity to chloramphenicol; however, no strain was resistant to chloramphenicol. Resistance to antibiotics in *Listeria* spp. is due to the acquisition genetic elements such as conjugative transposons, and self-transferable and mobilizable plasmids [37].

## Conclusions

In summary, the present study showed the prevalence of *L. monocytogenes* and other species in raw camel meat. All species and strains of *Listeria* exhibited high susceptibility to antibiotics in comparison with many resistance ratios and with studies on *Listeria* species isolated from other kind of meat or meat products. These may be due to the use of natural grasslands in feeding of camels, which is distributed throughout Saudi Arabia.

## Abbreviations

API: analytical profile index
BHI: brain heart infusion
PCR: polymease chain reaction
USDA: U.S.Department of Agriculture
